# Health-Related Quality of Life and Mental Health of Parents of Children with Pediatric Abdominal Tumors

**DOI:** 10.3390/children11080998

**Published:** 2024-08-16

**Authors:** Kira Zierke, Michael Boettcher, Paulina Behrendt, Safiullah Najem, Holger Zapf, Konrad Reinshagen, Wilhelm Wößmann, Johannes Boettcher

**Affiliations:** 1 Department of Pediatric Surgery, University Medical Center Hamburg-Eppendorf, Martinistrasse 52, 20246 Hamburg, Germany; 2 Department of Pediatric Surgery, University Medical Center Mannheim, University Heidelberg, Theodor-Kutzner-Ufer 1-3, 68167 Mannheim, Germany; 3 Department of Pediatric and Adolescent Surgery, Medical University of Vienna, Währinger Gürtel 18-20, 1090 Vienna, Austria; 4 Department of Child and Adolescent Psychiatry, Psychosomatics and Psychotherapy, University Medical Center Hamburg-Eppendorf, Martinistrasse 52, 20246 Hamburg, Germany; 5 Department of Pediatric Hematology and Oncology, University Medical Center Hamburg-Eppendorf, Martinistrasse 52, 20246 Hamburg, Germany; wi.woessmann@uke.de

**Keywords:** health-related quality of life, mental health, rare diseases, pediatric surgery, parents, pediatric abdominal tumors

## Abstract

Background: Abdominal tumors rarely occur in childhood but are associated with great psychological stress for patients and their families. Parents playing a central role in their children’s treatment may experience adverse effects on their Health-Related Quality of Life (HRQoL) and mental health due to the children’s illness and required treatment. Given the limited knowledge of the psychosocial burden of parents with children suffering from solid abdominal tumors, this study aims to assess their HRQoL and mental health along with the impact of various psychosocial factors. Methods: A cross-sectional cohort study was carried out involving 54 parents of children with solid abdominal tumors who had surgery at the University Medical Center Hamburg-Eppendorf in Germany. The results were compared with 46 parents of children undergoing routine surgeries without a prior tumor diagnosis, and with normative values. Data were obtained using standardized questionnaires. Results: Parents of the index group showed significantly worse HRQoL and limited mental health. Furthermore, they showed significantly higher parental burden in several subscales and less social support in comparison with the control group. Conclusions: Considering the lower parental HRQoL and the central role parents play in their children’s lives, it is crucial to include prevention and intervention measures for parents as part of a multimodal treatment regime for children with solid pediatric abdominal tumors.

## 1. Introduction

The diagnosis of a childhood tumor is a rare but very impactful experience that affects the entire life of a child and its parents [1]. While possible adverse psychosocial effects of pediatric tumors on survivors have been thoroughly studied in some research [2,3], very few studies assess their lasting effects on the mental health and HRQoL of parents with children with rare diseases [4,5]. However, according to our knowledge, there is a lack of analysis of parental HRQoL and mental health for solid embryonal abdominal tumors.

By definition, the prevalence of rare diseases in Europe is less than 5 out of 10,000 [6]. Due to their low prevalence, pediatric solid embryonal abdominal tumors belong to this category, with neuroblastoma and nephroblastoma appearing most frequently, closely followed by hepatoblastoma [7].

These tumors typically cause pain as a result of their extensive mass effect and expansion of the abdominal cavity [8]. Neuroblastomas are the most lethal solid extracranial tumors, as in approximately 70% of affected children, the cancer has already metastasized at the time of diagnosis [9]. Nevertheless, improved treatment options for children with cancer have led to an increasing number of children surviving their tumors and needing increased parental care at home [10]. While various treatment options, such as surgery, chemotherapy, and radiation, lead to successful treatment outcomes, they also involve various side effects and toxicity [11]. Psychosocial distress, include immunosuppression resulting in increased risk of infection, thrombocytopenia, anemia, malnutrition, mucositis, nausea with vomiting, and pain are some of the most frequent complications of cancer therapy [12]. In addition, chemotherapy can lead to serious long-term consequences, such as cardiovascular dysfunction, pulmonary dysfunction, renal dysfunction, hepatopathy, hearing loss, subsequent malignancies, and peripheral neuropathy [13]. This may result in a need for outpatient follow-up examinations, special therapies, and outpatient support services.

For parents, treatment options are accompanied by fears of possible death of the child or possible late effects, as well as conflict between work and care and increased financial burden [14].

The tremendous psychological and physical challenge of taking care of a child suffering from cancer at home may increase the risk of lower Health-Related Quality of Life (HRQoL) and deterioration of parents’ mental health [15].

The concept of HRQoL encompasses the assessment of the implications of a disease or health condition on a person’s physical health, psychological and social functioning, and mental well-being [16]. HRQoL describes a person’s self-perceived ability to actively partake in both physical and social activities and evaluates the degree of satisfaction and enjoyment they experience despite their illness or health condition [17]. Mental health defines “a state of well-being in which the individual realizes his or her own abilities, can cope with the normal stresses of life, can work productively and fruitfully, and is able to make a contribution to his or her community” [18] (pp. 231–233). As such, it plays an essential part in each individual’s development [19].

The association among psychosocial factors, HRQoL, and mental health can be derived from the biopsychosocial model [20]. The model considers interactions between genetic susceptibility, individual personality, the general social environment, and events stressful to the individual [21]. Within this framework, the social environment proves to be a central factor in coping with psychological challenges. Studies emphasize perceived social support as a potentially protective element that can mitigate the adverse effects of stress [22,23]. This dynamic is particularly evident in significant associations with positive mental health [24,25]. According to the biopsychosocial model, stressful events, such as a child’s tumor diagnosis and associated stressors including hospitalization and home care for the child [26], represent predispositions to reduced HRQoL and mental health.

Accordingly, previous research indicated that parents with children suffering from rare diseases experience more stress and loneliness [27] and report a reduced HRQoL in comparison to parents with healthy children [28]. In addition, studies suggest that the mental health of parents, particularly mothers, of children diagnosed with cancer is impaired [29]. This is evident through heightened feelings of insecurity, anxiety, and depression, especially in the early years after diagnosis [30,31].

Because parental well-being, which also has a fundamental influence on the HRQoL and psychosocial adjustment of children with cancer [32], is significantly stressed by the child’s diagnosis and treatment, a better understanding of the specific challenges and their impact is needed to adequately address the psychosocial requirements of parents and caregivers.

However, although previous studies provide evidence that parents have a significantly higher risk of reduced HQRoL and mental health than a healthy control group, the studies to date are insufficient to offer a complete understanding of the mental health and HRQoL of parents of children with solid abdominal tumors. Thus, the following research questions were investigated: (1) Are there any discrepancies in the distribution of HRQoL and mental health among parents of children with solid embryonal abdominal tumors, parents of children undergoing routine surgical procedures, and normative samples? (2) Are there specific subscales in which the index group differs from the control group and normative samples? (3) How do sociodemographic characteristics and psychosocial factors such as parental burden and social support predict the HRQoL and mental health of parents? (4) Are there differences between mothers and fathers in terms of their outcomes and predictors?

We expected the HRQoL of the index group to be below and the mental health of impacted parents to be more impaired than the control group and normative samples. Moreover, we expected parents in the index group to show greater impairment, particularly in the depression and anxiety subscales. In addition, we expected that non-disease-specific factors, such as the existence of care level, higher parental burden, and little social support, have a negative influence on HRQoL and mental health. In particular, we expected maternal HRQoL and mental health to be affected by negative predictors. The study offers insights into particular problematic aspects within relevant constructs and determines whether parents of children with solid abdominal tumors are susceptible to compromised HRQoL and mental health.

## 2. Materials and Methods

### 2.1. Study Design

The following research was designed as an observational cross-sectional study. Two predefined cohorts were compared using measurement of various parameters, assessed with validated questionnaires. The biopsychosocial model was used to test hypotheses about correlations or differences within the investigated groups. The index group is defined as children diagnosed with a pediatric solid abdominal tumor who underwent surgery treatment between 2011 and 2021, and their parents. The control group consisted of parents of children who underwent a routine surgery with no previous tumor diagnosis. The participants were identified and selected from the surgical registry of the Department of Pediatric Surgery at the University Medical Center Hamburg-Eppendorf in Germany. Additionally, data from normative populations were used to establish a comparison point for assessing the outcome variables in relation to the parental population. These normative data were derived from previous research that evaluated health outcomes in populations across different demographics, which are described below in the respective instruments.

The two groups were recruited between February 2022 and April 2023. The research approach builds on two previous studies conducted at the University Medical Center Hamburg-Eppendorf between April 2020 and November 2023. One examined the quality of life and mental health of children with rare diseases and their parents during the COVID-19 pandemic, and the other examined the health-related quality of life and mental health of children with embryonal abdominal tumors [3,5]. Ethical approval was granted by the Medical Chamber Hamburg (PV7161) and the study was preregistered at ClinicalTrials.gov (NCT05245123).

### 2.2. Variables and Instruments

The primary outcome of the survey was the assessment of parental HRQoL and mental health. The secondary aims were to identify psychosocial factors that might impact HRQoL and mental health. For this purpose, the following instruments were used.

**Parental Health-Related Quality of Life:** The European Quality of Life-5 Dimension (EQ-5D) was used to assess parental HRQoL [33]. The EQ-5D consists of a visual analog scale with scores from 0 to 100, where a higher score indicates better health status. Five subscales, each represented by one item, include mobility, self-care, general activities, pain/discomfort, and anxiety/depression. All of the five dimensions contain three possible response levels, whereby higher scores indicate a greater HRQoL. Using standardized calculation specifications, the results of the five questions are converted into an index value. This one-dimensional index expresses the respondent’s health status from 0 (very poor) to 1 (best possible) [33]. It has been shown that the EQ5D is one of the most widely used instruments for measuring HRQoL, and therefore provides normative data [34] and shows good validity and reliability [35].

**Parental mental health:** The Brief Symptom Inventory-18 (BSI-18) was used to assess parental mental health [36]. It is a shortened edition of the multidimensional versions of the Symptom Checklist 90-R, developed to capture a wide range of psychological problems. The BSI-18 includes the Global Severity Index (GSI), which includes each of the 18 items. GSI ratings can vary between 0 to 72, with higher scores indicating elevated psychological distress. The BSI-18 is proven to have reliable psychometric qualities with good to excellent internal consistency and normative data for the general German population split by gender [37].

The Patient Health Questionnaire (PHQ-9) was used to assess the severity of parental depression [38]. The PHQ-9 is a nine-item questionnaire that was developed as a screening method for the presence of depression. The scoring scale ranges from 0 to 27, and each item can be assessed on a four-point scale between 0 and 3. Increased scores are associated with greater impairment. Major depression is diagnosed when five or more of the nine criteria for depressive symptoms have occurred on at minimum half of the days at score 2 in the last two weeks, and one of the symptoms is depressed mood or anhedonia [39]. It has been shown that the unidimensional PHQ-9 has reliable and valid psychometric properties for assessing depression in the general population, which provides normative data [38].

The Generalized Anxiety Disorder Scale 7 (GAD-7) was used to assess the severity of parental anxiety [40]. This questionnaire provides information about the presence of GAD symptoms listed in the DSM-IV-Criteria. It contains seven items, of which each can take values from 0–3. Thus, the total score varies from 0 to 21, with heightened scores associated with elevated impairment. The GAD-7 has been shown to be an efficient and accurate self-assessment tool for anxiety [41] and provides population-based normative data that provide a framework for interpreting and comparing the prevalence of generalized anxiety disorder among different populations [40].

**Parental burden:** The German Version of the Impact on Family Scale (IoFS) was used to assess the family impact of the health condition [42]. The questionnaire contains 33 items that are answered on a 4-item scale. The 33 items form five subscales that assess overall negative impact on parents’ social life, personal stress, concern for possible siblings, financial impact, and coping problems. Furthermore, a total score based on all items can be calculated. Higher scores are associated with increased stress [42]. The instrument has demonstrated reliable psychometric properties with acceptable construct validity and good internal consistency, making it a valuable and user-friendly instrument for evaluating parental distress [43].

**Social support:** The Oslo Social Support Scale (OSSS-3) was used to assess parents’ social support level [23]. The questionnaire consists of three items that inquire about their amount of close confidants, feelings of concern for others, and relationship with acquaintances, focusing on the accessibility of practical support. Scores vary within a range of 3 to 14, wherein a score of 3 to 8 indicates low, 9 to 11 medium, and 12 to 14 high levels of social support [23]. The widely used OSSS-3 has proven to be a reliable and valid tool [25].

**Socio-demographic and clinical variables:** Participating parents were asked to fill out questions that collected demographic data such as gender, age, highest educational level completed, family structure, living situation, and employment status. Clinical variables included the children’s cancer type, date of the last surgical procedure, patient level of care, comorbidities, and possible ongoing psychotherapeutic treatment.

### 2.3. Participants

**Index group:** The following inclusion criteria were defined for the index group: (1) patient’s age < 18 years, (2) diagnosis of an abdominal tumor, including hepatoblastoma, neuroblastoma, nephroblastoma, or rhabdoid tumor, (3) surgical treatment at the University Medical Center Hamburg-Eppendorf, and (4) ability and willingness to complete questionnaires. Persons with major physical or cognitive disabilities were excluded from the study as participation was considered unreasonable. Prior to enrollment in the study, medical personnel identified all potential participants and reviewed diagnoses. Attendees received comprehensive verbal and written information about the study. In addition, the patient’s parents signed a declaration of consent to participate in the study. All participants could withdraw from the survey anytime.

Between 2011 and 2021, 111 children were treated due to a pediatric solid abdominal tumor at the Department of Pediatric Surgery. From this group of 111 potential participants, 12 were already deceased, nine did not have embryonal tumors, and ten were excluded due to a lack of contact information. Accordingly, 80 families were contacted by telephone in February 2022, informed about the study, and asked if they wished to participate. Since five immediately declined, questionnaires were sent out by post to 75 families in March 2022.

Out of 75 possible participating parents of children with a solid embryonal abdominal tumor, 54 completed the questionnaires. In 19 (35.2%), both parents sent back their answers, while 29 (53.7%) were only answered by the mother and 4 (7.4%) by the father. Two questionnaires (3.7%) were only answered by the parents about their children.

**Figure 1:**
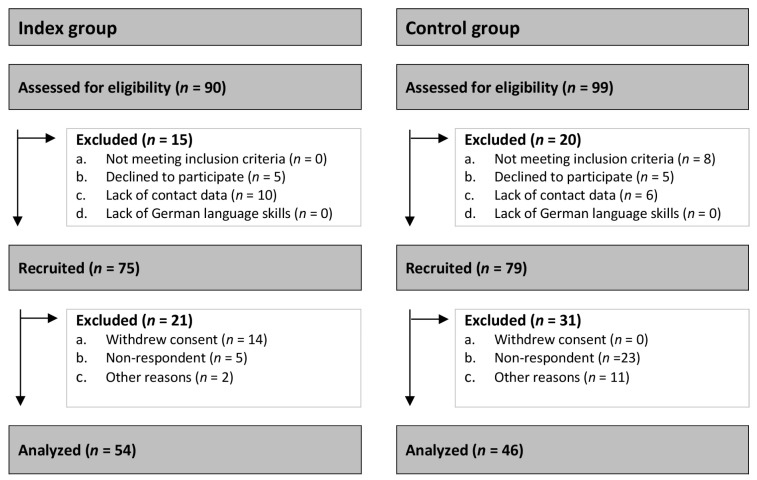
CONSORT flow diagram.

**Control group:** The following inclusion criteria were defined for the control group: (1) Parents of children < 18 years, (2) who underwent routine minor surgery at the department of pediatric surgery at University Medical Center Hamburg Eppendorf, (3) without a primary tumor diagnosis or any other congenital chronic disease, and (4) ability and willingness to complete questionnaires. In total, 91 families met the participation criteria during the period from September 2022 to April 2023. Five families did not want to participate in the study, so 86 families received the questionnaire. In total, we received 46 completed questionnaires back. Of these, 25 (54.3%) were answered by both parents, 18 (39.1%) from only the mother and 3 (6.6%) from only the father. Further details on the CONSORT flow diagram are provided in Figure 1.

### 2.4. Statistics

Frequencies, means, and standard deviation were used for descriptive analysis. Welch’s *t*-test for independent samples was employed to investigate potential differences between the index and control groups. One-sample *t*-tests were used to assess differences between the index group and normative reference values. To determine effect sizes Cohen’s d was calculated. Pearson correlations were applied to examine bivariate associations within the psychosocial outcome. Moreover, multiple linear regression models were conducted to identify predictors of psychosocial outcomes. Adjusted R^2^ served as an indicator of effect size. *p* < 0.05 (two-sided) was set as the statistical significance level. All statistical analyses were conducted with SPSS Statistics 29.

## 3. Results

### 3.1. Characterstics of the Study Population

Table 1 illustrates the sociodemographic and disease characteristics of the index and control group.

Concerning age of child, there was a small difference between participants (M =  8.45, SD =  3.26) and non-participants (M = 7.6, SD =  3.05) within the index group (*p* = 0.293, d = 0.266). However, no difference in the child’s gender was found between participants and non-participants (Cramer’s V = 0.019, *p* = 0.867). In terms of tumor entity, neuroblastoma was the most common diagnosis in the participating groups and nephroblastoma in the non-participating groups (Cramer’s V = 0.249, *p* = 0.206).

When comparing the index and control groups, the socio-demographic data of the two groups were overall comparable. Regarding the age of the participants, no relevant discrepancies were found between the index and control groups for mothers and children. The fathers in the index group were younger than the fathers in the control group, with an average difference of five years. In addition, there were no differences regarding gender, marital status, number of children, parental employment, or parental educational level.

In terms of children, there were more girls in the index group, while more boys were included in the control group. Children in the index group often had a significantly greater level of care than children in the control group. The most common surgical indications among children from the index group were neuroblastoma and nephroblastoma, followed by hepatoblastoma and rhabdoid tumor. In contrast, in the control group, the most common reason for minor pediatric surgical treatment was bone fracture, followed by other causes.

### 3.2. Differences in Psychosocial Variables between Parents of Children with Solid Abdominal Tumor, Control Group and Normative Samples

Table 2 shows the self-assessed HRQoL and mental health scores, along with the values for social support and stress, of parents in the index and control groups and from normative samples.

In comparison to the control group and normative samples, significantly lower HRQoL scores were found for both mothers and fathers in the index group. With regard to the HRQoL subscales, significant differences were observed between the index and control groups in mobility, habitual activity, pain, and discomfort in the maternal assessment. Meanwhile, the paternal evaluation showed a significant difference in the subscales of feeling anxiety and depression. Effect sizes varied between moderate and large. Compared to normative data, mothers in the index group reported significantly fewer problems in terms of mobility and usual activities. However, both parents in the index group reported significantly more problems on the anxiety and depression subscales. Again, effect sizes varied between moderate and large.

Overall, the mental health of parents in the index group also showed increased values in comparison to mothers and fathers of the control group and normative samples, but without significance. The situation was similar to the scores across the three subscales, except for the somatization scale, in which mothers in the index group had significantly higher values than mothers in the control group. The effect sizes varied between small and large. In terms of depressive symptoms, parents of the index group had worse values than the control group, albeit without significance and with a small effect size. Compared to normative samples, mothers in the index group demonstrated significantly different results with small effect sizes. Regarding generalized anxiety symptoms and social support, no relevant differences were found between the groups.

When comparing parental burden, all outcome variables showed higher values in the index group compared to the control group. This was equally true for maternal and paternal scores. Significantly higher values were reported by mothers and fathers of the index group in the subscales of personal and daily social stress and burden of sibling children. Fathers additionally had a significantly higher total stress score, which was not the case for mothers. Effect sizes varied between small and large. A more detailed examination of each subscale with associated outcome variables can be found in Supplementary Tables S1–S4.

### 3.3. Correlation Analyses

Table 3 illustrates the Pearson correlation coefficients and the extent of potential associations between parental HRQoL and mental health and individual predictors of psychosocial outcomes, separated by gender. Significant bivariate associations were found between the level of care variable and HRQoL for both mothers and fathers in the index group and between social support, mental health, and HRQoL. In addition, a significant association was observed between age of the child and time since the initial surgery for both parents. Mothers additionally exhibited a significant correlation with level of care, amount of social support, and family burden. The extent of the associations ranged from weak to strong.

### 3.4. Predictors for Psychosocial Outcomes in Parents of Children with Solid Abdominal Tumors

Table 4 shows multiple regression models examining the child’s age, time since initial surgery, and level of care, as well as social support and parental burden, as potential predictors of psychosocial outcomes in parents of children suffering from solid abdominal tumors. Analysis demonstrated that higher levels of care were associated with decreased maternal HRQoL, while higher levels of social support were significantly associated with improved maternal HRQoL. The overall model was able to explain 13.3% of the variation in HRQoL reported by the mothers. In terms of maternal mental health, older age of the child, the presence of a level of care, and increased parental burden were associated with decreased mental health, while increased distance from initial surgery was associated with better mental health. Increased social support led to significantly better maternal-reported mental health. The overall model was found to be significant and could explain 17% of the variance in mental health reported by the mothers.

For paternal reported HRQoL, the presence of a level of care was associated with decreased and higher social support with increased HRQoL. Increased age of the child was linked with significantly lower HRQoL, while increased distance from initial surgery led to significantly better paternal HRQoL. The overall model proved to be statistically significant and was able to explain 62.6% of the variation in the fathers’ results. Concerning the fathers’ reported mental health, the presence of a level of care, increased parental burden, and older age of the child were related to decreased mental health. In contrast, an increasing time distance from the child’s initial surgery and increased social support were associated with better paternal-reported mental health. The overall model provided an explanation for 41.4% of the variance in paternal mental health.

## 4. Discussion

In the present study, we investigated HRQoL and mental health in mothers and fathers of children with solid embryonal abdominal tumors. The study results indicate that parents of children with solid embryonal abdominal tumors have significantly poorer HRQoL compared to control and normative samples. In-depth analysis revealed gender-specific differences in the factors predicting parental HRQoL and mental health.

It has been shown that pediatric solid embryonal abdominal tumors have the potential to considerably affect the lives of patients and their families. Due to medical advances, the amount of parents taking care of a chronically diseased child is increasing [44]. Long-term consequences of the disease and extensive therapies place a heavy burden on both the affected children and their families [44,45]. While there is increasing research on the psychosocial health of children with solid tumors, the parents of these children, despite their central caregiving role, remain an often-overlooked group [28].

The study results show that parents of the index group have significantly worse HRQoL than the control group and existing normative samples, with significantly larger effect sizes compared to normative samples. This is consistent with other study results that have shown that parents of chronically diseased children have significantly decreased HRQoL compared to parents with healthy children [44,46] and indicates that there is a gap remaining in healthcare where it comes to addressing the psychosocial needs of parents with chronically sick children, especially those with solid embryonal abdominal tumors.

Our study revealed that mothers in the index group reported more difficulties in mobility, usual activities, and pain and discomfort. Meanwhile, fathers experienced significantly more frequent feelings of anxiety and depressive symptoms. These differences indicate that future support programs should be adapted to the individual burdens of parents.

The control group also exhibited lower HRQoL than normative samples. This finding may be related to the fact that our control group consists of participants from the outpatient clinic. Previous research has demonstrated that hospitalization during childhood inevitably causes stress for the entire family, potentially resulting in a decline in HRQoL and mental health [47]. In accordance with the biopsychological model, it is plausible to assume that hospitalization, in general, can lead to an impaired HRQoL.

Regarding mental health, contrary to our expectations, no significant differences were observed between the index and control groups, nor were these found in comparison with normative samples. These findings differ from other studies [5,48,49]. The results suggest that professional and comprehensive care provided by staff specializing in the treatment and care of children with solid embryonal abdominal tumors may have a significant impact on the mental health of caring parents. In addition, it may be possible that ongoing improvements in therapeutic options, which are associated with increased life expectancy of children [50], also contribute to an increase in the psychological well-being of parents. At this point, it should be mentioned that deceased children were excluded from participation.

Nevertheless, the outcomes indicated slightly higher psychological distress among parents in the index group. Both parents displayed heightened restlessness, with mothers additionally exhibiting elevated signs of anxiety and irritability. These gender discrepancies in parental mental health align with current literature [51] and the prevailing notion that mothers often take on the role of primary caregiver, leading to potential mental health implications [28]. However, it should also be noted that we collected more than twice as much data from mothers than fathers in the index group. Consequently, further research is required to explore the paternal caregiving role and its effect on mental health.

Our ultimate goal was to identify predictors for the psychosocial outcome variables of parents with children suffering from solid embryonal tumors. In contrast to a previous study [52], no significant link could be demonstrated between the age of surviving children and their mother’s well-being. However, an increased age of the child was associated with significantly worse paternal HRQoL. The same applies to longer periods elapsed since the initial surgery of the child. For both parents, a longer time since the first operation was a positive predictor of mental health, but only fathers showed a significant association between longer time and better HRQoL. Conversely, the child’s level of care proved to be a decisive predictor of parental HRQoL and mental health. This is in line with existing research findings that indicate that greater physical strain as a result of an increased caregiving burden is associated with reduced HRQoL and impaired mental health [5]. This could indicate that parents whose children receive a level of care should be given more attention in the future.

Increased social support, in contrast, was identified as a significant positive predictor of better psychosocial results among both parents. This is consistent with the results of other studies [46,48] showing that a good support system is essential for the well-being of parents. Regarding prevention, parents should be encouraged to maintain their social network and be offered appropriate support services.

In conclusion, it was shown that higher parental burden was associated with poorer HRQoL, particularly for fathers in the index group. Consistent with our expectations, parents in the index group reported significantly higher family stress on several subscales, such as daily social and personal burdens, as well as the burden of concern for siblings. The comprehensive care required for a child demands a big amount of parents’ time and leaves them with limited freedom [53]. It can be inferred that this results in high levels of parental stress. This is in line with other research findings [54,55], which have additionally shown that increased parental stress levels also have a negative impact on children’s emotional health and development [55]. Reducing stress is a possible starting point for interventions to strengthen the mental health of both parents and children.

## 5. Study Limitations

To evaluate the study, the following limitations should be considered: (1) The time difference in recruitment of index and control groups. (2) The rarity of the disease and the corresponding small study population. (3) All families were recruited at the University Medical Center Hamburg-Eppendorf in Germany. Thus, the study is monocentric, and to transfer the results to other hospitals and countries, a multicenter global study is recommended. (4) There is a possibility of nonresponse bias among families from whom we did not receive responses. It is possible that participating parents generally have better HRQoL and mental health than non-participating parents. (5) Given the treatment period from 2012 to 2022, differences in diagnostic approaches and treatments between groups could impact the observed variability in HRQoL and mental health.

## 6. Conclusions

Parents with a child affected by a solid embryonal abdominal tumor reported significantly worse HRQoL but equal mental health compared with the control group and the normative samples.

Due to the lower parental HRQoL, prevention and intervention measures targeting mothers and fathers should be included in the treatment plan for a child with solid embryonal abdominal tumors. This is especially relevant for parents with high stress, little social support, and whose child has a level of care. Possible interventions to improve parental well-being would include reinforcing personal resources and referrals to support services such as support groups in the community. As only minor differences regarding mental health were demonstrated within the index and control groups, future research should focus on this aspect. A new treatment program focused on family support has recently been introduced in Germany and is now undergoing evaluation in a multicenter randomized controlled trial [56].

Given the limited amount of studies on parental HRQoL and mental health in children with rare diseases [56] like pediatric solid embryonal abdominal tumors, further investigations should not only address high-prevalence diseases but also much rarer ones. Furthermore, it would be useful to examine gender differences to adjust interventions for gender where appropriate, as well as to look at a time course of HRQoL and mental health rather than just collecting cross-sectional studies.

## Figures and Tables

**Table 1:** Sociodemographic and disease characteristics in the index and control groups.

	Index Group (a) (*n* = 54 Families)		Control Group (b) (*n* = 46 Families)		Differences a vs. b
Characteristics	*M*	*SD*	*M*	*SD*	*p*
Patient’s age (years)	8.45	3.3	9.51	4.48	0.201
Mother’s age (years)	41.39	4.95	40.28	6.78	0.444
Father’s age (years)	42.81	3.2	47.13	6.79	**0.021**
Number of children mothers	2.13	1.18	2.07	0.75	0.795
Number of children fathers	2.14	1.46	2.24	1.02	0.775
Time since initial diagnosis (years)	5.50	2.54	*-*	-	
Time since first surgery (years)	5.37	2.54	*-*	-	
**Parents**	*n*	%	*n*	%	
Parent’s gender (mothers/ fathers)	48/22	88.9/40.1	43/29	93.5/63.0	
Marital status (mothers/ fathers)					0.737/0.478
Married/ Living together	40/20	83.3/91.0	34/26	79.0/89.6	
Single	6/1	12.5/4.5	6/1	14.0/3.4	
Divorced	2/1	4.2/4.5	3/1	7.0/3.4	
Not stated		0.0/0.0	0/1	0.0/3.4	
Education (mothers/ fathers)					0.712/0.942
Lower-middle education	17/9	35.4/40.9	15/11	34.9/37.9	
Higher education	29/12	60.4/54.6	26/16	60.5/55.2	
Not stated	2/1	4.2/4.5	2/2	4.6/6.9	
Employment ^1^ (mothers/ fathers)					0.139/0.666
Fully employed	7/17	14.6/77.3	14/25	32.6/86.2	
Partly employed	29/5	60.4/22.7	22/3	51.2/10.3	
No employment	4/0	8.3/0.0	6/1	13.9/3.5	
Not stated	8/0	16.7/0.0	1/0	2.3/0.0	
**Patients**	*n*	%	*n*	%	
Patient’s gender					**0.007**
Female	32	59.3	15	32.6	
Male	22	40.7	31	67.4	
Patient receives level of care ^2^					**<0.001**
Yes	22	46.3	4	8.7	
No	25	40.7	36	78.3	
No data	7	13.0	6	13.0	
Patient solid abdominal tumors					
Neuroblastoma	24	44.4	-	-	
Nephroblastoma	22	40.7	-	-	
Hepatoblastoma	6	11.1	-	-	
Rhabdoid Tumor	2	3.7	-	-	
Others	0	0	-	-	
Children control group diseases					
Hernia	-	-	3	6.5	
Fimosis	-	-	2	4.3	
Median Neck Cyst	-	-	0	0	
Bone Fracture	-	-	21	45.7	
Testicular Malposition	-	-	0	0	
Others	-	-	20	43.5	

Note. ^1^ Refers to the last 12 months. ^2^ Referes to the decision for the classification in the care insurance according to the German long-term care insurance.

**Table 2:** Distribution of parental HRQoL, mental health, social support and burden for the index group, the control group, and normative data according to the EQ5D, BSI, PHQ-9, GAD-7, OSSS-3, and IoFS.

	Index Group (a)		Control Group (b)		Normative Data (c)		Differencesa vs. b	Differencesa vs. c	Effect Sizea vs. b	Effect Size a vs. c
	*M*	*SD*	*M*	*SD*	*M*	*SD*	*p*	*p*	*d*	*d*
**Mothers**										
HRQoL (EQ5D Index Value)	0.83	0.13	0.88	0.10	0.93	0.11	**0.031**	**<0.001**	−0.448	−0.730
Mental health Total Score (BSI)	6.02	7.59	4.44	5.45	5.07	7.80	0.280	0.400	0.237	0.125
Depression(PHQ-9)	5.00	4.77	4.92	4.06	3.10	3.50	0.937	**0.010**	0.017	0.398
Anxiety (GAD-7)	5.02	4.27	4.10	3.65	4.07	3.53	0.285	0.141	0.232	0.223
Social Support (OSSS-3)	10.46	2.20	11.00	2.26	10.34	2.20	0.257	0.721	−0.244	0.053
Parental Burden (IoFS)	2.12	0.36	1.97	0.39	-	-	0.056	-	0.411	-
**Fathers**										
HRQoL (EQ5D Index Value)	0.83	0.15	0.90	0.06	0.94	0.53	**0.004**	**<0.001**	−0.600	−0.728
Mental health Total Score (BSI)	5.52	12.24	2.52	2.59	4.19	6.9	0.203	0.607	0.360	0.109
Depression (PHQ-9)	3.91	5.31	2.78	2.46	2.70	3.50	0.329	0.298	0.283	0.228
Anxiety (GAD-7)	3.09	4.31	2.32	2.11	3.01	3.12	0.411	0.932	0.233	0.018
Social Support (OSSS-3)	10.30	2.42	10.90	1.80	10.50	2.20	0.316	0.702	−0.283	−0.081
Parental Burden (IoFS)	2.20	0.47	1.80	0.34	-	-	**<0.001**	-	1.000	-

Note. HRQoL = Health-Related Quality of Life. EQ-5D = European Quality of Life 5 Dimensions 3 Level Version, BSI = Brief Symptom Inventory, PHQ-9 = Patient Health Questionnaire, GAD-7 = Generalized Anxiety Disorder Scale, OSSS-3 = Oslo social support scale, IoFS = Impact on Family Scale. Raw scores of the EQ5D have higher values corresponding to higher parental QoL. Raw scores of the BSI have increased values correlating with lower parental mental health. Comparison among groups was evaluated with Welch’s *t*-test and one-sample *t*-test. Effect size was determined using Cohen’s *d*.

**Table 3:** Pearson correlation coefficients and the level of potential associations between individual predictors for psychosocial outcomes.

Variables	1	2	3	4	5	6	7
**Mothers (*n* = 48)**							
1. Age of child	-						
2. Time since initial surgery	**0.560 (<0.001)**	-					
3. Level of care	**−0.346 (0.020)**	−0.274 (0.062)	-				
4. Social Support (OSSS-3)	0.109 (0.481)	**0.312 (0.035)**	**−0.345 (0.020)**	-			
5. Parental Burden (IoFS)	−0.243 (0.107)	−0.204 (0.169)	**0.349 (0.017)**	−0.137 (0.371)	-		
6. HRQoL (EQ-5D)	0.229 (0.130)	0.246 (0.096)	**−0.297 (0.038)**	**0.433 (0.003)**	−0.110 (.465)	-	
7. Mental Health (BSI-18)	-0.086 (0.581)	−0.287 (0.053)	0.269 (0.074)	**−0.517 (<0.001)**	0.121 (0.430)	**−0.703 (<0.001)**	-
*M*	8.38	5.46	0.44	10.46	2.12	0.83	6.02
*SD*	3.28	2.57	0.5	2.19	0.36	0.13	7.59
**Fathers (*n* = 22)**	**1**	**2**	**3**	**4**	**5**	**6**	**7**
1. Age of child	-						
2. Time since initial surgery	**0.739 (<0.001)**	-					
3. Level of care	−0.271 (0.310)	−0.203 (0.378)	-				
4. Social Support (OSSS-3)	−0.035 (0.895)	−0.133 (0.546)	−0.210 (0.362)	-			
5. Parental Burden (IoFS)	0.008 (0.975)	−0.119 (0.587)	0.275 (0.227)	−0.290 (0.180)	-		
6. HRQoL (EQ-5D)	−0.184 (0.480)	−0.151 (0.492)	**−0.429 (0.002)**	**0.592 (0.003)**	−0.248 (0.253)	-	
7. Mental Health (BSI-18)	0.085 (0.747)	0.199 (0.362)	0.408 (0.066)	**−0.672 (<0.001)**	0.347 (0.105)	**−0.899 (<0.001)**	-
*M*	8.06	5.17	0.4	10.3	2.2	0.83	5.52
*SD*	2.89	2.42	0.501	2.42	0.46	0.14	12.23

Note. *M*: Mean, *SD*: Standard deviation. Main entries are Pearson *r*, with *p* values in parentheses. HRQoL = Health Related Quality of Life. EQ-5D = European Quality of Life 5 Dimensions 3 Level Version, BSI = Brief Symptom Inventory. OSSS: Oslo Social Support Scale, IoFS: Impact on Family Scale.

**Table 4:** Prediction of psychosocial measures in parents of children with solid abdominal tumors.

	Constant			Age			Time since Initial Surgery			Level of Care				Social Support			Parental Burden			R^2^
	*b*	95% CI	*p*	*b*	95% CI	*p*	*b*	95% CI	*p*	*b*	95% CI	*p*	*b*	95% CI	*p*	*b*	95% CI	*p*	*b*	*p*
Mothers																				
HRQoL	**0.432**	**[0.026, 0.839]**	**0.038**	0.006	[−0.011, 0.022]	.502	−0.001	[−0.023, 0.021]	0.947	−0.026	[−0.128, 0.075]	0.599	**0.029**	**[0.006, 0.051]**	**0.013**	0.024	[−0.120, 0.167]	0.739	0.133	0.069
mental health	**24.082**	**[1.250, 46.914]**	**0.039**	0.280	[−0.646, 1.206]	.542	−0.529	[−1.795, 0.737]	0.402	1.125	[−4.504, 6.754]	0.687	**−1.596**	**[−2.922, −0.271]**	**0.020**	−0.313	[−8.241, 7.615]	0.937	**0.170**	**0.043**
Fathers																				
HRQoL	**0.709**	**[0.296, 1.122]**	**0.003**	**−0.043**	**[−0.076, 0.010]**	**0.015**	**0.039**	**[0.001, 0.077]**	**0.045**	**−0.251**	**[−0.376, −0.125]**	**0.001**	0.011	[−0.016, 0.038]	0.380	0.116	[−0.023, 0.255]	0.092	**0.626**	**0.008**
mental health	18.871	[−10.123, 47.865]	0.178	1.601	[−0.701, 3.902]	0.152	−2.090	[−4.768, 0.589]	0.113	8.135	[−0.675, 16.946]	0.067	−1.347	[−3.210, 0.516]	0.138	−1.808	[−11.568, 7.951]	0.688	0.414	0.059

Note. Bold values indicate statistical significance at the *p* < 0.05 level. CI = confidence interval. HRQoL = Health Related Quality of Life. Level of care: yes = 1, no = 0.

## Data Availability

The data presented in this study can be obtained from the corresponding author upon request. Due to privacy restrictions, the data are not publicly accessible.

## Supplementary Materials

The following supporting information can be downloaded at: https://www.mdpi.com/article/10.3390/children11080998/s1, Supplementary Table S1. Distribution of parental HRQoL for the index group and the control group of the five dimensions of the EQ5D; Supplementary Table S2. Distribution of parental mental health for the index group, the control group, and norm data of the BSI; Supplementary Table S3. Distribution of parental mental health for the index group, the control group, and norm data of the subscales of the GAD-7; Supplementary Table S4. Distribution of parental burden for the index and the control group of the IoFS.
