# Supplementary material for: Health-Related Quality of Life and Mental Health of Parents of Children with Pediatric Abdominal Tumors
Source: Children (Basel). 2024 Aug 16;11(8):998. doi: 10.3390/children11080998 (PMC11352779; doi:10.3390/children11080998)
Supplement: Supplementary file 1 [file children-11-00998-s001.zip › children-3154158-Supplementary Materials-final.pdf]

Supplementary Materials:

Supplementary Table S1. Distribution of parental HRQoL for the index group and the control group of the five dimensions of the EQ5D.

	Index group (a)		Control group (b)		Differences a vs. b	Effect size a vs. b
	M	*SD*	*M*	*SD*	*p*	*d*
**Mothers**						
Mobility	1.08	0.28	1.00	0.00	**0.044**	0.408
Self-care	1.02	0.15	1.00	0.00	0.323	0.204
Usual activity	1.08	0.28	1.00	0.00	**0.044**	0.408
Pain / Discomfort	1.42	0.54	1.10	0.30	**<0.001**	0.725
Anxiety / Depression	1.39	0.54	1.24	0.48	0.146	0.308
**Fathers**						
Mobility	1.13	0.34	1.00	0.00	0.083	0.565
Self-care	1.00	0.00	1.04	0.19	0.326	-0.255
Usual activity	1.08	0.29	1.00	0.00	0.162	0.450
Pain / Discomfort	1.39	0.50	1.21	0.42	0.182	0.388
Anxiety / Depression	1.35	0.65	1.04	0.19	**0.035**	0.674

Note: Comparison between groups is assessed with Welch *t*-test and one-sample *t*-test. Effect size was calculated by using Cohen's *d*.

Supplementary Table S2. Distribution of parental mental health for the index group, the control group, and normative data of the BSI.

	Index group (a)		Control group (b)		Normative data (c)		Differences a vs. b	Differences a vs. c	Effect size a vs. b	Effect size a vs. c
	M	*SD*	*M*	*SD*	*M*	*SD*	*p*	*p*	*d*	*d*
**Mothers**										
1) Somatization	1.74	2.63	0.78	1.53	1.54	2.70	**0.035**	0.596	0.443	0.078
2) Depression	1.83	2.90	1.79	2.42	1.92	3.30	0.933	0.837	0.018	-0.030
3) Anxiety	2.28	3.30	1.98	2.42	1.59	2.70	0.624	0.161	0.103	0.208
**Fathers**										
1) Somatization	1.83	3.88	0.59	0.95	1.36	2.40	0.147	0.570	0.464	0.120
2) Depression	2.30	5.24	0.79	1.32	1.56	3.10	189	*0*.502	0.419	0.142
3) Anxiety	1.39	3.51	1.14	1.46	1.26	2.40	0.748	0.859	0.098	0.037

Note: BSI = Brief Symptom Inventory. Raw scores of the BSI, with higher values corresponding to lower parental mental health. Comparison between groups is assessed with Welch *t*-test and one-sample *t*-test. Effect size was calculated by using Cohen's *d*.

Supplementary Table S3. Distribution of parental mental health for the index group, the control group, and normative data of the subscales of the GAD-7.

	Index group (a)		Control group (b)		Normative data (c)		Differences a vs. b	Differences a vs. c	Effect size a vs. b	Effect size a vs. c
	M	*SD*	*M*	*SD*	*M*	*SD*	*p*	*p*	*d*	*d*
**Mothers**										
Feeling nervous	0.63	0.65	0.56	0.71	0.64	0.71	0.635	0.920	0.103	-0.015
Not able to stop worrying	0.42	0.68	0.39	0.63	0.44	0.66	0.850	0.813	0.040	-0.034
Worry about different things	0.71	0.74	0.71	0.78	0.65	0.70	0.995	0.589	0.001	0.079

Trouble relaxing	1.04	0.96	0.88	0.78	0.81	0.78	0.377	0.102	0.187	0.244
Being restless	0.60	0.79	0.29	0.64	0.50	0.72	**0.044**	0.367	0.429	0.132
Easily annoyed or irritable	0.94	0.78	0.83	0.59	0.65	0.64	0.459	**0.014**	0.155	0.367
Feeling afraid	0.67	0.69	0.44	0.59	0.36	0.61	0.103	**0.004**	0.350	0.442
**Fathers**										
Feeling nervous	0.30	0.70	0.29	0.46	0.41	0.61	0.914	0.479	0.032	-0.150
Not able to stop worrying	0.39	0.78	0.07	0.26	0.30	0.56	0.072	0.582	0.572	0.117
Worry about different things	0.52	0.90	0.32	0.48	0.49	0.65	0.342	0.867	0.287	0.035
Trouble relaxing	0.61	0.89	0.57	0.57	0.57	0.68	0.863	0.837	0.051	0.043
Being restless	0.13	0.34	0.29	0.46	0.39	0.64	0.175	**0.002**	-0.377	-0.754
Easily annoyed or irritable	0.83	1.07	0.64	0.56	0.61	0.63	0.464	0.344	0.221	0.201
Feeling afraid	0.30	0.70	0.14	0.36	0.24	0.52	0.324	0.665	0.299	0.092

Note: Comparison between groups is assessed with Welch *t*-test and one-sample *t*-test. Effect size was calculated by using Cohen's *d*.

Supplementary Table S4. Distribution of parental burden for the index and the control group of the IoFS.

	**Index group (a)**		**Control group (b)**		**Differences a vs. b**	**Effect size a vs. b**
	M	*SD*	*M*	*SD*	*p*	*d*
**Mothers**						
Personal burden	1.88	0.65	1.43	0.56	**<0.001**	0.744
Daily social burden	1.77	0.47	1.49	0.60	**0.018**	0.525
Financial burden	1.73	0.80	1.71	0.63	0.864	0.036
Coping Problems	3.38	0.64	3.20	0.79	0.257	0.260
Burden of siblings	1.55	0.51	1.31	0.49	**0.047**	0.478
**Fathers**						
Personal burden	1.97	0.60	1.21	0.29	**<0.001**	1.652
Daily social burden	1.75	0.55	1.29	0.27	**<0.001**	1.114
Financial burden	1.72	0.83	1.48	0.51	0.244	0.349
Coping Problems	3.36	0.78	3.20	0.79	0.237	0.203
Burden of siblings	1.57	0.42	1.10	0.22	**<0.001**	1.431

Note: Comparison between groups is assessed with Welch *t*-test and one-sample *t*-test. Effect size was calculated by using Cohen's *d*.
